# Bridging the Digital Divide in Diabetes Care With an AI-Powered Health Coaching Intervention: Qualitative Formative Study

**DOI:** 10.2196/81441

**Published:** 2026-08-07

**Authors:** Tejossy John, Sangeetha Mohanraj, Tanjila Nawshin, Tapan Mehta, Mohanraj Thirumalai

**Affiliations:** 1 Department of Orthopedic Surgery, Heersink School of Medicine, University of Alabama at Birmingham, Birmingham, AL, United States; 2 Department of Family and Community Medicine, Heersink School of Medicine, University of Alabama at Birmingham, Birmingham, AL, United States; 3 Research Collaborative, School of Health Professions, University of Alabama at Birmingham, Birmingham, AL, United States; 4 Department of Health Services Administration, School of Health Professions, University of Alabama at Birmingham, Birmingham, AL, United States; 5 Division of General Internal Medicine and Population Health, Heersink School of Medicine, University of Alabama at Birmingham, 701 19th Street South, ALGEN 580, Birmingham, AL, 35294, United States, 1 312 218 7740

**Keywords:** type 2 diabetes, digital health coaching, patient education, diabetes self-management, patient experience, qualitative research, phenomenology, diabetes technology, expectations

## Abstract

**Background:**

Despite advancements in digital health coaching (DHC) for type 2 diabetes self-management, a persistent digital divide limits access among underserved populations, necessitating low-tech, telephone-based alternatives.

**Objective:**

This formative qualitative study explored the lived experiences of type 2 diabetes mellitus (T2DM) self-management challenges among adults in Alabama and elicited preferences for telephone-based DHC to inform intervention optimization.

**Methods:**

This study was part of a larger National Institutes of Health clinical trial (5R01DK129378) that examined the feasibility and needs of a telephone-based DHC intervention for self-management of T2DM. The study employed a qualitative, phenomenological approach to assess patients’ needs surrounding digital coaching. Twelve patients diagnosed with T2DM were recruited between August and December 2022. Data collection involved in-depth, semistructured interviews conducted via telephone calls or secure Zoom sessions. All interviews were audio-recorded, professionally transcribed, and verified for accuracy. Two independent coders analyzed the data using NVivo software version 14 Plus.

**Results:**

Two themes emerged: (1) multifaceted barriers to diabetes self-management spanning behavioral (dietary adherence and nutritional gaps), physical/environmental (mobility limitations and weather constraints), and structural domains (medication shortages, insurance coverage limiting continuous glucose monitoring); (2) expectations for telephone-based DHC, including targeted education (meal planning, exercise adaptations, and medication effects), health coaches as accountability partners, daily reminders and check-ins via texts/calls, and incentives as extrinsic motivators to adherence. Insights from the study directly shaped the intervention components of a pilot feasibility trial (NCT05344859).

**Conclusions:**

Key challenges in day-to-day T2DM management included diet, physical activity, medication unavailability, and lack of insurance coverage for continuous glucose monitors. Expectations for a potential telephone-based DHC intervention included targeted education on diet, exercise, and medication effects; perceptions of health coaches as accountability partners; reminders via texts/calls as beneficial; and monetary incentives as extrinsic motivators for adherence to the intervention.

## Introduction

Type 2 diabetes mellitus (T2DM), a chronic metabolic disorder characterized by insulin resistance and impaired glucose regulation, presents a significant public health concern, imposing a substantial burden on individual well-being and the health care system in the United States. According to the National Diabetes Statistics Report published by the Centers for Disease Control and Prevention, approximately 38.4 million individuals are currently struggling to manage uncontrolled diabetes. Concurrently, an estimated 8.7 million adults remain undiagnosed with the condition [1].

Primary care physicians (PCPs) play a central role in diabetes management, delivering care to approximately 90% of individuals diagnosed with the condition amid a nationwide shortage of board-certified endocrinologists [2-4]. PCPs assume multifaceted responsibilities for early detection, routine monitoring, treatment planning, and lifestyle counseling [5,6]. However, high patient volumes and limited consultation time contribute to a substantial workload burden. The median primary care visit lasts only 15.7 minutes and typically addresses 6 distinct clinical issues [7], constraining the capacity to deliver comprehensive diabetes care [8-10].

People with T2DM face numerous daily challenges, including a significant need for lifestyle modification [11,12], consistent medication adherence [11], and psychosocial difficulties [13,14], adapting to physical symptoms and management of comorbidities [15-17]. Digital health coaching (DHC) emerged as an evidence-based intervention to alleviate the burden on PCPs and enhance patient outcomes. DHC applies behavioral science, clinical strategies, and skillful conversation to promote sustainable health behavior change, thereby improving diabetes self-management, fostering lifestyle changes, and enhancing clinical outcomes [18-20].

Over the past decade, as smartphone use has surged dramatically [21], this proliferation has paralleled a marked rise in smartphone-based and technology-enabled remote health coaching interventions, shifting traditional care delivery toward scalable, patient-centered models that leverage mobile apps and telehealth platforms. Numerous studies have substantiated the efficacy of digitally powered health coaching in diabetes management, consistently demonstrating clinically meaningful reductions in HbA_1c_ levels [22-26]. Such evidence underscores the potential of digital coaching to address gaps in primary care capacity while empowering patients with real-time support and personalized guidance; however, a digital divide limits equitable access for underserved populations.

However, the penetration of digital technologies, particularly smartphones, continues to expand across the United States [27]. This growth remains uneven and is accompanied by persistent disparities stratified by age, gender, education, and income levels [27]. Beyond material access, such as internet connectivity and smartphone access, many individuals face a second level of digital divide in the skills needed to use these technologies effectively. Trends reported by Hong and Cho [28] on older adults and digital health interventions highlight these challenges and recommend developing senior-friendly resources to overcome age-related barriers to technology adoption and usability. Similarly, a study by Kumar et al [29] highlighted that health interventions that use advanced mobile features may widen disparities by excluding those with low mobile literacy. Simple mobile phone capabilities, such as text messaging, require significant health literacy and have previously affected enrollment [30] and adoption [31] of texting-based interventions.

To bridge the digital divide, we proposed a study design—an intervention delivered via the simplest widely available technology, a telephone call—without requiring internet access, smartphones, or digital skills, thereby ensuring broad inclusivity for underserved populations while remaining fully compatible with modern smartphones for those who prefer or possess them. This exploratory qualitative study was structured as a comprehensive needs assessment to understand patients’ priorities, unmet needs, and expectations regarding a telephone-based diabetes self-management intervention. By systematically gathering qualitative insights from a small sample of the target population, we sought to ensure that the intervention design aligned with real-world patient experiences and preferences. This patient-centered approach directly guided the design and development of the interactive voice response (IVR)–supported telephone coaching intervention and was conducted as a formative substudy within the larger NIDDK (National Institute of Diabetes and Digestive and Kidney Diseases)-funded clinical trial (5R01DK129378), which evaluated the feasibility and effectiveness of an AI-powered telephone-based DHC intervention for T2DM management among adults in the Deep South [32].

This study aims to expand the foundational knowledge about patients’ lived experiences with T2DM by understanding two key areas: (1) self-management burden, including daily challenges encountered during T2DM self-management, and (2) expectations from telephone-based interventions, including participants’ perceptions and preferences for a future DHC platform delivered via phone and compatible technologies. Insights from this formative work were used to develop a user-centric, culturally tailored telephone and IVR-based health-coaching intervention, subsequently operationalized in the GODART (Gamified Optimized Diabetes Management with Artificial Intelligence–Powered Rural Telehealth) IVR tracking and goal-setting system, optimized for adults with T2DM, and designed to be accessible to those facing technological or socioeconomic barriers in the Deep South of the United States.

## Methods

### Study Design

This exploratory qualitative study employed semistructured interviews and phenomenological thematic analysis as a formative needs assessment to understand patients’ challenges and expectations for a telephone-based DHC intervention for T2DM self-management. Semistructured interviews were conducted with a purposive sample of patients with T2DM. The resulting data were analyzed using rigorous, systematic qualitative methods, including thematic analysis and intercoder agreement assessment [33].

### Recruitment and Sample

Purposive sampling was conducted among the residents of the state of Alabama who were (1) clinically diagnosed with T2DM, (2) aged 18 years or older, (3) able to converse in English, and (4) had access to a phone. Patient recruitment occurred through the University of Alabama at Birmingham (UAB) Health System. Clinical staff approached patients who visited the Family and Community Medicine Clinic to determine their willingness to participate in a semistructured qualitative interview. Recruitment for this study took place between August and December 2022.

Patients who expressed interest were contacted over the phone by the research team to confirm eligibility and obtain verbal consent. Interviews were then scheduled over the telephone or in a closed Zoom conferencing room for approximately 45 minutes. During the interview, the study staff followed the semistructured interview guide to elicit and record participant responses (Multimedia Appendix 1). The purpose of the interview was to gain an in-depth understanding of the lived experiences and challenges of patients with T2DM living in Alabama, as well as their expectations regarding a telephone-based DHC intervention. Afterward, the audio recordings were transcribed via a professional transcription service, Rev, Inc [34], and thematic analysis was conducted on the resulting transcripts. Data saturation was achieved after 12 semistructured interviews, as no new themes or insights emerged from the last 2 participants. This sample size aligns with established qualitative phenomenological research standards and captures a comprehensive range of patient experiences and expectations related to T2DM management and DHC in this population [35].

### Ethical Considerations

The study was approved as exempt by the UAB Institutional Review Board under protocol number IRB-300008277. Verbal informed consent was obtained from all participants during the screening call. To protect privacy and ensure confidentiality, all participant data were deidentified. Participants were compensated US $25 for participating in the interview.

### Data Analysis

#### Analytic Approach

Our basic data analytic approach was exploratory and content-driven [36]. The rationale for choosing an exploratory approach was that it provided an opportunity to inductively expand the knowledge base on patients’ experiences with T2DM, the challenges they face in managing the disease, and patients’ expectations of a telephone-based DHC program for managing T2DM [37]. The specific approach to analysis was phenomenology because this approach uses people’s subjective experiences to gain an in-depth understanding of a phenomenon [38]. Since we explored the challenges patients face in managing T2DM and how those lived experiences shaped their expectations surrounding a telephone-based DHC program, the phenomenological approach appeared to be a good fit for the study. We followed the data analysis steps outlined in the Creswell and Creswell [33] guidelines. We first organized and prepared the transcripts for analysis by transcribing the audio-recorded files.

#### Development of Codebook

After an initial immersion in the transcripts, the 2 coders independently read all transcripts multiple times and wrote analytic memos to capture their initial impressions. We conducted both the first and second cycles of coding.

First-cycle coding was conducted line-by-line using a combination of descriptive codes (summarizing segments of data) and in vivo codes (participants’ own words). Each transcript was initially coded independently by 2 researchers to identify participants’ concrete experiences with diabetes self-management and expectations for telephone-based coaching.

After coding the first set of transcripts, the coders met to compare their code lists, collapsed overlapping codes, and agreed on common code labels and definitions, which formed the first version of the codebook. This codebook was iteratively refined as additional transcripts were coded; new codes were added when data segments did not fit existing codes, while similar codes were merged or refined to improve clarity and consistency. Each codebook revision was discussed and agreed upon by both coders, and discrepancies were resolved through discussion, with a third qualitative expert consulted when consensus could not be reached.

In the second coding cycle, the team used pattern coding to group the first cycle of codes into higher-order categories or themes that reflected broader concepts (eg, combining multiple diet-related codes into a broader category reflecting diet management) [39].

These categories were then systematically compared across transcripts to identify recurring patterns and relationships. These categories were further abstracted into themes and subthemes that described patterns across participants. Candidate themes were continuously checked against the coded data and raw transcripts to ensure they were grounded in participants’ accounts. Negative or divergent cases that did not fit initial interpretations were examined. Themes were finalized only when all coders agreed that they comprehensively captured the dataset’s range of perspectives.

#### Intercoder Reliability and Rigor

Two researchers independently coded all transcripts, and assistance from a third coder was sought when the first 2 coders could not reach an agreement on coding. The intercoder agreement level was calculated using the Huberman method [39], and an agreement level of >90% was observed. NVivo software version 12 Plus (Lumivero) was used to organize, code, and categorize the information obtained from the transcripts.

A substantial amount of time was spent on the analysis, and expert help was sought when necessary. Deidentified data were used throughout the analysis. An analytic perspective was maintained throughout the analysis, using deidentified data while remaining empathetic to participants’ life experiences [40]. Opinions from all participants were given equal weight when interpreting the data to avoid selective interpretation [40]. It was ensured that the data remained fully confidential, and only the researchers involved in this study had access to it [39].

#### Research Team and Positionality

The analytical team consisted of 2 UAB-based researchers, one with expertise in public health and the other in health services research with prior qualitative research experience, who collaboratively coded the transcripts. Both researchers had long-standing experience working with adults with T2DM in the Deep South, including predominantly African American, socioeconomically disadvantaged populations receiving care within the UAB Health System [32,41,42]. To ensure credible analysis, a third qualitative expert, a health service researcher with extensive experience in qualitative studies, was consulted in case of coding disagreement or interpretive uncertainty.

## Results

### Participants and Eligibility Criteria

A total of 12 participants completed semistructured interviews for this study. Their demographic characteristics are summarized in Table 1. Participants were predominantly female (n=10, 83.33%) and African American (n=10, 83.33%), with a mean age of 54.6 (SD 12.39) years, and most had lived with diabetes for 5 to 10 years (n=7, 58.56%). Most participants (n=7, 58.33%) had a high school diploma or equivalency, 3 (25%) had less than high school education, and 2 (16.66%) held bachelor’s degrees. The patient Area Deprivation Index (ADI) was categorized as low (ADI value 1‐3), medium (ADI value 4‐7), and high (ADI value 7‐10). The distribution across ADI categories showed that 6 (50%) participants resided in high-deprivation areas, 5 (41.67%) in medium-deprivation areas, and 1 (8.33%) in low-deprivation areas.

**Table 1.** Demographic information (N=12).

Patient	Age (y)	Gender	Race	Education	Duration since diagnosis (y)	Area Deprivation Index
P1	59	Female	African American	Less than high school degree	18	7
P2	67	Female	White	High school graduate or GED^a^	1.3	6
P3	73	Female	African American	High school graduate or GED	11	7
P4	32	Male	African American	High school graduate or GED	5	9
P5	53	Female	African American	Bachelor’s degree	3	1
P6	67	Male	African American	Less than high school degree	5	10
P7	45	Female	African American	High school graduate or GED	4	10
P8	64	Female	African American	High school graduate or GED	7	5
P9	34	Female	African American	High school graduate or GED	9	5
P10	43	Female	African American	High school graduate or GED	10	8
P11	57	Female	African American	Bachelor’s degree	10	9
P12	38	Female	White and Hispanic	Less than high school degree	8	9

a GED: General Educational Development.

All participants had confirmed a diagnosis of T2DM for at least 1 year, ranging between 15 months and 18 years. Each participant self-reported multiple comorbid conditions, including, but not limited to, circulatory issues (eg, hypertension), metabolic system disorders (eg, hypercholesterolemia), and diagnoses related to their mental health (eg, anxiety disorder).

Some other reported comorbidities included respiratory conditions (eg, asthma and chronic obstructive pulmonary disease), liver conditions (not specified), allergies, anemia, migraines, sleep difficulties, etc.

Two themes, each with several subthemes, emerged from the analysis of 12 transcripts. The first theme demonstrated the multifaceted challenges participants face in diabetes self-management, and the second theme explored the participants’ anticipation of a telephone-based DHC intervention for diabetes management. Each theme and its related subthemes are described in detail in the following section.

### Theme 1. Challenges to Diabetes Self-Management

#### Theme Overview

Diabetes self-management presents persistent, multifaceted challenges that test patients’ knowledge, resources, and resilience daily. Participants in this study expressed challenges across four key domains, including (1) managing diet, (2) physical activity, (3) medication access barriers, and (4) blood glucose monitoring (Table 2).

**Table 2.** Participant-reported challenges to diabetes self-management.

Domain	Subthemes	Description	Quotes
Behavioral	Managing diet	Struggles with dietary adherence, cravings, and lack of nutritional literacy	“Well, when I first was told that I had diabetes, it was kind of hard for me to eat different foods, because I was scared, I was eating the wrong thing… I don’t know how to go in the grocery store and read on the label, cholesterol this, or this much this, that, and another…” (P8) “Basically, eating sweet stuff.. that’s very hard, but I try to manage it. If I do eat it, I just take a little bit of more insulin.” (P9) “A lot of times I don’t feel like cooking anything, so I’ll just maybe eat some cereal or maybe drink a Boost in the morning.” (P3) “Yes. It’s very difficult to me. When you work, that’s a little harder because you can’t always eat six meals a day.” (P5) “My challenges that I have, is years of eating a certain way and doing a certain thing, and your mindset of thinking that you’re Superman and nothing can hurt you. Now I’m finding out that I have high blood pressure and diabetes, so I’m having to make a life change..” (P4)
Physical and environmental	Mobility constraints	Functional and environmental limitations that limit physical activity	“Well, problems I have been having since I had diabetes.. My leg’s been hurting…and it messed with my walking. It messed up my legs up. I can’t hardly walk sometimes.” (P7) “I have challenge with my ankles and stuff swelling up, and I sweat a lot.. So, it is difficult to walk” (P1) I do. I try to walk. I haven’t been doing that lately. (Because it is cold outside) (P11)
Structural	Medication and monitoring access	Systemic barriers, including unaffordable copays, medication stock-outs, and lack of insurance for CGM^a^ devices	“It goes up and down (referring to blood sugar levels). Because I don’t take no medicines... Since I was taking the shot once a week, and they went up on my diabetes (medication costs)….they went up on it, and my insurance did… and I couldn’t afford what they went to, cause I was paying a US $40 copay, and it went up to 196.” (P6) “It’s not that I’m forgetting. For the past three months, I’ve been having trouble getting my medication. Trulicity is never in stock, so she (primary care provider) changed it to, I don’t want to get the name wrong, but it starts with an O (referring to a new medication)… and that’s not in stock either. So the last doctor visit I had, she changed it to yet another medication and it is not covered on Medicaid… so I reached out to her on my portal to let her know… I don’t know what I’m going to receive now.” (P10) “I ran out of strips… when I had strips and everything… I did. I don’t do it no more. Because I know when it’s high, and I know when it’s low.” (P12) The Libre was what I was using. I’m not using anything right now. I had ran out… and I found out that the military doesn’t cover that… that’s why it’s taking me so long to get it back. (P4)

a CGM: continuous glucose monitoring.

#### Behavioral Barriers: Managing Diet

The most frequently reported challenges were related to dietary management. Participants described diet as a major challenge in managing diabetes in their daily lives (n=10). Many individuals struggled to resist ingrained eating habits and sweet cravings. While some mentioned that sweets were “getting around me” despite their efforts to limit intake (P12), others just compensated for it with an additional dose of insulin instead of practicing abstinence (P9). Participant (P11) reported having adequate nutritional knowledge but lacking appropriate behavioral execution, citing situational limitations on healthy options and a lack of willpower in choosing better alternatives. For some, knowledge gaps in interpreting food labels and the fear of eating incorrectly were concerns. Participant (P8) reported that the initial diagnosis of diabetes brought overwhelming confusion regarding grocery shopping, reading labels, and food selection. Additional concerns about managing diet included situational limitations such as a busy work schedule (P5) and struggles with daily meal planning (P3), which led to choosing convenient food over meeting nutritional requirements.

#### Physical and Environmental Barriers: Mobility Constraints

Physical activity was hindered by physical limitations, including low energy, fatigue, limb pain, weakness, and visual impairment, as well as by environmental factors for some participants (n=4). Diabetes-related complications, such as leg pain and weakness, impaired mobility (P7), and ankle swelling, further hindered ambulation (P1) for participants. Extreme climates also interfered with outdoor walking routines for some participants (n=3), as participant P11 mentions: “I try to walk. I haven’t been doing that lately. (Because it is cold outside).” Participants not limited by physical limitations reported engaging in some mild-to-moderate physical activity. Walking emerges as the most common and accessible form of exercise.

#### Structural Barriers: Medication Access and Monitoring

Medication adherence was generally good among the participants in this study; however, structural barriers disrupted treatment access for some individuals. Barriers related to medication access included the unavailability of prescribed drugs and lack of insurance coverage (n=3). These challenges resulted in delays or changes in prescribed regimens. The available and potentially alternative medicines either had high copays, were not affordable for patients, or were not covered by insurance at all. Such experiences highlight financial barriers, insurance limitations, and medication shortages, leading to disturbances in adherence, even in highly motivated individuals. In these situations, participants often relied on health care providers for alternative solutions.

Glucose monitoring also emerged as an essential component of diabetes self-management for most participants, who incorporated blood glucose checks into their daily routines using traditional glucometers or continuous glucose monitoring (CGM) systems. However, structural barriers similarly affected access to monitoring tools (n=3). Challenges included supply shortages and insurance-related limitations on obtaining glucometers or CGM devices. Some participants expressed difficulty maintaining regular monitoring due to a lack of supplies or discomfort with fingerstick testing. Participant P12 discontinued fingerstick testing after exhausting test strips and now relies on symptom recognition for hyperglycemia or hypoglycemia. Participant P4 faced insurance barriers to receiving CGM monitors and mentioned that they are in the process of resolving access issues.

#### Theme Summary

Overall, the findings highlight that diabetes self-management is influenced by a complex interplay of behavioral, physical, and structural factors. Participants faced persistent difficulties in adhering to dietary recommendations, engaging in consistent physical activity, accessing and affording prescribed medications, and maintaining regular blood glucose monitoring. These challenges were compounded by long-standing behaviors, physical comorbidities, environmental constraints, and systemic barriers within health care and insurance systems. The convergence of these individual and contextual factors underscores the multifaceted nature of diabetes self-management and reveals the necessity for comprehensive, patient-centered interventions that address not only education and motivation but also accessibility, affordability, and continuity of care.

### Theme 2. Future Interventions: Expectations Surrounding Telephone-Based DHC for Diabetes Management

#### Theme Overview

The multifaceted nature of diabetes self-management necessitated understanding patients’ needs and expectations for an efficient, personalized telephone-based DHC program. Four subthemes emerged within this theme: (1) targeted education, (2) perception of a health coach, (3) delivery modality, and (4) incentive option. A brief description of each subtheme is provided in Table 3.

**Table 3.** Future interventions: expectations surrounding telephone-based digital health coaching for diabetes management.

Subthemes	Description	Quotes
Targeted education	What to eat Specific exercises Day-to-day regime Medication effect All about diabetes None Guidance on specific topics such as meal planning, exercises, and medication side effects	“I need to know more about my varieties, …I was in question about sweet potatoes and white potatoes. I didn’t really get a good clarification whether I can eat those two or not, and different other things.” (P3) “I would like to know what specifically exercises that I can do if I ain’t doing the right ones.” (P7) “I would like to know before they start... Okay. Say, for instance, they give you injections. Because before I was on injections, I was on pills. So I would like for the doctor or whomever to talk to you more about what the injections can do to your body or what it may make you feel like once you don’t have it because I didn’t start to feel like the drain and all of that until I wasn’t able to take my meds.” (P10)
Perception of a health coach	Accountability partner to provide feedback and tailored meal preparation strategies	“I learn best by hands-on.… if I’m talking to somebody that’s telling me, "Okay, this is what I want you to do. You can actually print this out and this is your breakfast, this is your lunch, this is your dinner and snacks,” and so on and so forth. And I can go shopping and get these things and actually pre-prepare my meals and things like that.” (P4) “I think it’ll keep me... help me make better choices, keep me informed about what’s out there, and what’s available for me to control my diabetes.” (P11) “Of course. Well, just having someone to hold you accountable is always a good thing. Someone you can ask questions to about certain things would always benefit me.” (P5)
Delivery modality	Prefer text reminders with options for short, high-impact calls	“Sometimes I forget, I have to ask my husband did he see me take my medicine because sometimes I forget and I’d be like, “Okay, I don’t know if I remembered to take it. Did I take it or did I not?” So I think it [daily call/text] would help. That would be beneficiary helping me.” (P9) “Just to make sure you’re doing it to stay on track, and make sure you’re doing what you’re supposed to do. I think it [weekly calls] would be vital. I think it’d be a help.” (P11)
Incentive options	Monetary rewards are mainly preferred, while some prefer gym memberships	“… I think it would help me a lot financially. Because I’m on a fixed income, and I just got a prescription that I need to get filled. … The money would come in handy.” (P8) “Probably a membership to a gym or something [should be appropriate reward].” (P10) “I’m not really worried about that [reward]. I’m more so worried about my health.” (P3)

#### Targeted Education

Based on their experiences with current T2DM management strategies, the participants were asked whether they would like to incorporate specific knowledge into future T2DM interventions to help them monitor and manage their diabetes. Participants expressed targeted interests in diabetes education topics, with dietary guidance emerging as the most common need (n=3), particularly clarification on suitable foods for individuals with diabetes, as P7 mentions, “And then I can see about my eating habits, too, see what I can eat….what I can’t eat...I know a little of it, but I don’t know a lot of it.” Participants also emphasized the need to learn day-to-day regimens to build sustainable habits (n=2), noting the difficulty of changing long-standing lifestyles and the value of clear daily guidance for effective habit formation. Additional knowledge on other topics included specific exercises (n=1), side effects of diabetes medication (n=1), and the desire for overall refreshers (n=3). However, some indicated no need for additional information (n=4), citing self-learning, prior knowledge, or prior participation in research studies as sufficient.

Upon querying participants’ thoughts on receiving a customized booklet containing diabetes education information, accompanied by a follow-up call to explain its contents, most participants expressed interest in learning more and felt it would be a valuable tool (n=11). They mentioned that the booklet would serve as a quick, convenient guide for finding any information they needed compared to searching online on an electronic device.

#### Perceptions of a Health Coach

The concept of a health coach was introduced to the participants as a personal cheerleader, a guide, and a partner who could help them implement lifestyle changes to improve their health. When asked, the participants were asked whether they thought they would benefit from consulting with a health coach to manage diabetes. Most participants (n=8) were open to working with a health coach and expressed interest in tailored behavioral support. A few (n=2) were unsure of the idea and were willing to try if recommended, while only a couple (n=2) declined the idea of working with a coach, citing sufficient self-control and an aversion to added stress. Most participants perceived two primary benefits of working with a health coach: (1) an accountability partner for informed decision-making and keeping them on the right track and (2) providing support on personalized meal planning.

Participants also mentioned that receiving health coaching would help them develop strategies to manage their diabetes. They perceived that communicating information about their diabetes management to a health coach would help them receive constructive feedback, which, in turn, would enable them to manage their diabetes more effectively.

#### Delivery Modality: Use of Technology in Terms of Phone Calls or Text

Participants perceived using technology, such as a phone call or text, as part of the intervention would be vital.

Participants thought daily monitoring texts or phone calls could be helpful reminders because they had memory issues (n=10). They mentioned having difficulty remembering to take medications on time or checking their blood sugar levels. When asked about their preferred communication mode, participants preferred text over calls (n=7). Participants who favored texts did not like the idea of regular phone calls, as P9 quoted, “Probably a text because I don’t really like phone calls, so I’d rather text than take a phone call.” Participants who opted for calls (n=5) preferred quick interactions, as P10 stated, “It would be preferably a call because text messages, I’m not going to lie, they’re easy for me to ignore. So yeah.” Although most participants preferred receiving a daily text over a daily call, we also asked participants about the desired length of a daily call if they were to receive one; most specified that they wanted it to be short. The majority of participants preferred the call duration to be less than 10 minutes (<2 minutes [n=4], 5‐10 minutes [n=4], as per the needs of the participants [n=1]).

Participants were also asked about their preference for weekly contact for coaching calls. Most participants (n=11) indicated that these calls would be a helpful component of the intervention, providing accountability and reminders to support routine building. One participant preferred a reduced frequency (once or twice a month), suggesting that weekly calls might be too repetitive and frustrating (P1).

#### Incentive Structure

Participants identified practical incentives to enhance engagement with daily calls or texts, with monetary rewards emerging as the predominant preference (n=10). Most favored financial support through cash or gift cards, with monetary rewards ranging from US $10 to US $50, viewing it as valuable supplemental income. P8 mentions: “...it would help me a lot financially... The money would come in handy.” A minority prioritized health over rewards (n=2), with P3 stating: “I’m not really worried about that [reward]. I’m more so worried about my health,” and P10 preferring alternatives that promote healthy behavior, such as a gym membership.

#### Theme Summary

Participants described their expectations surrounding a potential DHC intervention within this theme. Given the challenges they face in managing diabetes, they expected future interventions to incorporate specific information related to diet, day-to-day regimens, exercise, and medication effects to optimally manage their diabetes. Most participants identified several aspects of health coaching that would benefit them in managing diabetes. They perceived that a health coach could serve as an accountability partner, a guide for meal planning, and assistance in staying on track with diabetes management, while also providing a means to discuss aspects of diabetes beyond their PCP. Most participants found that using technology, such as daily or weekly calls and texts, would help them as reminders to take medications or exercise, except for a few who indicated that daily or weekly communications could be overwhelming. Most participants stated that a monetary incentive of US $10 to US $50 would motivate them to attend those calls, while a few noted that a financial incentive might not be as effective as other incentives, such as a gym membership.

## Discussion

### Principal Findings and Implications for Telephone-Based Digital Health Coaching

This qualitative study explored the lived experiences of individuals with T2DM, focusing on the daily challenges faced in achieving optimal glycemic control. It also examined participants’ perceptions of a prospective health coaching intervention aimed at facilitating effective diabetes self-management and promoting long-term glycemic stability. The challenges identified among participants were consistent with findings from previous studies, highlighting the interplay of behavioral, physical, environmental, and structural factors that collectively impede effective diabetes self-management [43-45]. Insights and needs assessment findings from this study informed the conceptualization, design, and development of an AI-assisted telephone-based health-coaching intervention GODART pilot and feasibility trial (NCT05344859) to address the self-management gaps identified by participants [32]. Informed by participants’ preferences for accessible, personalized support, the GODART intervention delivered daily IVR calls via an automated behavior-monitoring system to track diet, physical activity, medication adherence, and blood glucose monitoring, along with weekly coaching.

The persistent difficulties participants described with self-management of T2DM included adhering to dietary recommendations, integrating regular physical activity into daily routines, affording and accessing medications, and sustaining consistent blood glucose monitoring. These challenges were often rooted in long-standing eating patterns [46,47], limited nutritional literacy [48-51], physical limitations [52,53] and fatigue [54-56], environmental barriers to exercise [53,57,58], insurance-related constraints [59], and intermittent access to monitoring supplies or continuous glucose monitoring devices [60]. Taken together, the results underscore that diabetes self-management is not simply a matter of individual motivation but is embedded within broader socioeconomic, physical, and health system constraints that complicate efforts to maintain optimal glycemic control [61-64].

Participants expressed a strong interest in a future telephone-based DHC program aimed at providing targeted diabetes education and bridging the practical and informational gap. They articulated a desire for nuanced, personalized guidance on what to eat, how to interpret food labels, how to choose safe and realistic forms of physical activity given their functional limitations, and how diet, exercise, and medications interact to affect blood sugar. They also expressed a desire for clear guidance on building day-to-day routines that could help them overcome long-standing habits and develop sustainable, long-term lifestyle changes. This finding aligns with evidence highlighting the importance of personalized, practical information that addresses patients’ immediate challenges and promotes adherence to self-management practices [65-67]. The emphasis on day-to-day routines suggests that participants value straightforward, actionable education over general advice [67-69]. Such person-centered approaches have been shown to improve comprehension, motivation, and glycemic control, especially when they align with individuals’ health literacy levels and behavioral readiness [70-72].

We also found an overwhelmingly positive attitude toward the idea of receiving a customized diabetes education booklet. All participants unanimously viewed it as a valuable tool for learning more about diabetes, serving both as an overall refresher and a practical guide for managing diabetes [73,74]. This enthusiastic response highlights the importance of providing educational resources that are concise, visually engaging, and tailored to individuals’ literacy levels [75]. Traditional diabetes education materials, which often contain dense or highly technical content, frequently fail to capture patients’ attention or promote sustained learning [75-78]. In contrast, developing simplified, low-literacy educational packets may be more effective for improving understanding and facilitating long-term behavioral change among adults with type 2 diabetes [74,77,79]. In response, the GODART study developed a pictorial, low-literacy educational packet that incorporated materials from the Diabetes Literacy and Numeracy Education Toolkit [80] and the Centers for Disease Control and Prevention’s National Diabetes Prevention Program [81] to ensure accessible, consistent foundational knowledge for all participants.

Our participants described a health coach as a nonjudgmental, accountable partner and a guide who helps improve health and implement lifestyle changes [82,83]. Most participants expressed a clear preference for working with a health coach to support behavior change, while a few were uncertain but willing to try one if recommended, suggesting that participants were generally open to modifying their behaviors when adequate support was available [19,84]. The effectiveness of health coaching programs appears greater when they are delivered more frequently within long-term interventions [20,85,86]. Frequent check-ins, typically held weekly or bi-weekly [87,88], enable timely adjustments to SMART (specific, measurable, attainable, realistic, and timely) goals based on participants’ real-world challenges and help reduce overall stress levels [89-92]. Based on these responses, all participants in the GODART study received 24 weekly personalized health-coaching calls that guided them in setting progressive, achievable milestones for sustainable behavior change.

Regular behavior monitoring via texts, calls, or apps has been shown to enhance accountability and awareness in diabetes management for medication adherence, blood glucose monitoring, dietary behavior, and physical activity [93-96]. Echoing this evidence, most participants endorsed the idea that daily reminders, such as text messages or phone calls, may help them remember to take their medications on time and check their blood sugar as recommended. Only one participant expressed reluctance toward behavior monitoring via texts, calls, or apps due to concerns about frustration and repetition of the calls [97]. In response, we developed an automated behavior-monitoring system delivering daily IVR calls using AI-assisted natural language processing for the GODART study, where participants reported daily dietary intake, step counts, exercise duration, medication adherence, and glucose monitoring captured via natural speech, keypad inputs for numeric data, and simple yes or no responses [98-100]. To address call-related frustrations, participants selected their preferred call times and had flexible return-call options, making it low burden while supporting consistent engagement [97].

Participants identified incentives as strong motivators for engaging with DHC texts or calls [101,102]. Preferences varied, with most participants reporting that monetary rewards were particularly motivating, while others emphasized nonmonetary incentives or intrinsic motivation related to health improvement. The diversity in motivational drivers is consistent with the existing literature, which shows that financial incentives can enhance short-term engagement and adherence to health interventions, particularly among individuals facing socioeconomic challenges [101,103]. Additionally, although both monetary and nonmonetary incentives were motivating, financial rewards appeared to exert a stronger influence than psychological motivators [104]. Based on this feedback, the GODART study incorporated financial incentives of up to US $100.80 to encourage adherence to daily monitoring calls over a 24-week intervention period.

Although participants’ challenges with diet, physical activity, and structural barriers to diabetes self-management largely mirror existing qualitative literature, this formative study makes 2 key contributions. First, it focuses specifically on expectations for a low-tech, telephone-based DHC model intended to bridge the digital divide for adults with T2DM who have limited broadband access, smartphone use, or mobile literacy. Second, the study demonstrates how participant-identified needs, such as targeted diabetes education, health coaches as accountability partners, weekly coaching check-ins, low-literacy educational materials, daily reminders, and financial incentives, can be translated into concrete components of a telephone-based, AI-assisted IVR intervention. By systematically integrating and prioritizing patient perspectives over researcher-driven assumptions, this formative qualitative work directly informed the design of the GODART optimization and feasibility trial, illustrating how qualitative inquiry can be operationalized into a scalable, equity-oriented DHC intervention.

### Limitations

The study’s findings are based on a purposively selected sample from a single health system in the Deep South state of Alabama, which may limit the generalizability of the results to a broader population with T2DM in the Deep South. The sample size of this exploratory study was relatively small (n=12), and participant demographics were relatively homogeneous across age, race/ethnicity, socioeconomic status, and educational attainment. This homogeneity may have increased the risk of selection bias and limits the breadth of perspectives captured, potentially overlooking key topics, barriers, or preferences relevant to underrepresented subgroups—such as rural residents, diverse ethnicities, or individuals with varying levels of health literacy.

All participants were recruited from a single academic health system, the UAB, and were required to have access to a phone and be able to speak English to participate in the interview. This requirement potentially excluded individuals with limited access to health care or digital technology, as well as those from non–English-speaking backgrounds. Additionally, the study relied on self-reported data from semistructured interviews, which may be subject to recall and social desirability bias, as participants might have overreported or underreported certain behaviors or attitudes. Finally, while the study provides valuable insights into patient expectations for DHC, it does not assess the actual implementation or effectiveness of such interventions. Future research should include larger, more diverse samples and employ longitudinal or mixed methods approaches to better understand the evolving needs and outcomes of individuals with T2DM, particularly as digital health solutions continue to expand.

### Conclusion

Our findings highlight the multifaceted daily challenges adults with T2DM face in achieving glycemic control. The study also reveals participants’ strong interest in accessible, personalized, telephone-based health coaching interventions as a supportive tool for managing their condition through lifestyle counseling and psychosocial support. By translating participant insights into the design of the GODART pilot and feasibility trial, including low-literacy education, AI-assisted daily behavior monitoring, human vs automated coaching, and fixed vs adaptive incentives, this research exemplifies patient-centered optimization within the MOST (Multiphase Optimization Strategy) framework. These findings not only affirm the feasibility of scalable digital health solutions but also underscore the critical role of preparatory qualitative work in bridging self-management gaps to foster sustainable behavior change and long-term health equity. Future studies should build on this foundation to evaluate effectiveness across diverse settings and refine components for broader implementation.

## Supplementary material

10.2196/81441 Multimedia Appendix 1 Interview guide.

## Abbreviations

ADI: Area Deprivation Index
CGM: continuous glucose monitoring
DHC: digital health coaching
GODART: Gamified Optimized Diabetes Management with Artificial Intelligence–Powered Rural Telehealth
IVR: interactive voice response
MOST: Multiphase Optimization Strategy
NIDDK: National Institute of Diabetes and Digestive and Kidney Diseases
PCP: primary care provider
SMART: specific, measurable, attainable, realistic, and timely
T2DM: type 2 diabetes mellitus
UAB: University of Alabama at Birmingham
