# Supplementary material for: Bridging the Digital Divide in Diabetes Care With an AI-Powered Health Coaching Intervention: Qualitative Formative Study
Source: JMIR Diabetes. 2026 Aug 7;11:e81441. doi: 10.2196/81441 (PMC13450810; doi:10.2196/81441)
Supplement: Supplementary file 1 [file diabetes-v11-e81441-s001.docx]

*Hello, my name is ______________________. Thanks for agreeing to participate in this interview and we are thankful for your time. I would like to ask you a series of questions. There are no right or wrong answers. If any question or details I provide are not clear, feel free to ask for clarifications or more information. If you do not feel comfortable answering any question, simply let know. Okay?*

*[I will not refer you by name to protect your privacy. Do I have the permission to record this call?]*

***[******start recording********]***

# For People with Diabetes

**Section A: Introduction**

1. How long have you had diabetes?
2. Are there any other health conditions you have and for how long?
3. Can you tell me about things you do to manage your diabetes? [prong for daily blood sugar measurement, medicines, diet and exercise]
4. Can you talk about the challenges you face when trying to manage diabetes and health? [prong for daily blood sugar measurement, medicines, diet and exercise]
5. Have you found a way to handle these challenges? [prong for daily blood sugar measurement, medicines, diet and exercise]

**Section B: Perceptions About Interventions In General**

6. Are there specific topics about diabetes management you would like to know more about?
7. Do you have primary care provider?

*If yes,*

a. *How does your interaction with your primary care provider help with your diabetes management?*
b. *Have you ever been referred to an endocrinologist or other services that help monitor your diabetes?*

*If no PCP, why do you not have a primary care provider?*

8. What do you use to check your diabetes in your home? If you do not, why not? What are the barriers? If yes, frequency.

*Next, I would like to talk about health coaches. A health coach is a personal health cheerleader, guide and partner. They could help people implement lifestyle changes to improve their health.*

9. Do you think you would benefit from the help of a health coach to help manage your diabetes? If yes, how? If no, why?
10. Do you think you would benefit from technologies to remind you about and track your healthy behaviors for managing your diabetes? If yes, can you describe how can technology be used? If no, why?

**Section C: Questions Specific to our Proposed Intervention**

*We are creating a diabetes management program that uses health coaches and automated phone calls to help manage diabetes. We would like to hear to some of your thoughts to help us design this program. We have a few specific questions for this and would like your response.*

**Baseline Education**

11. Do you think a customized printed booklet sent to your home and followed up with a call from a health coach would be a satisfactory approach to ensure that you have the required knowledge about diabetes?

**Daily Monitoring**

12. Do you think a daily quick call / text message to you to check on your diabetes management would be helpful? If yes, how? If no, why? Which would be your preferred mode of communication with a health coach (text vs call)? For phone calls, what would be your desired call length?

**Rewards**

13. If we were to reward you for simply answering our quick calls to monitor your diabetes management, do you think that it would motivate you to respond to the call? If yes, what would be an appropriate reward?

**Coaching Calls**

14. Do you think a weekly call from a health coach to discuss about the challenges you faced over the week and strategies to overcome them would help? If so, how? If not, why?

**[****Thank you for your participation in this interview **********]**
